# Supplementary material for: Reader engagement with medical content on Wikipedia
Source: eLife. 2020 Mar 6;9:e52426. doi: 10.7554/eLife.52426 (PMC7089765; doi:10.7554/eLife.52426)
Supplement: Transparent reporting form [file elife-52426-transrepform.docx]

***eLife’s* transparent reporting form**

We encourage authors to provide detailed information *within their submission* to facilitate the interpretation and replication of experiments. Authors can upload supporting documentation to indicate the use of appropriate reporting guidelines for health-related research (see [EQUATOR Network](http://www.equator-network.org/%20)), life science research (see the [BioSharing Information Resource](https://biosharing.org/)), or the [ARRIVE guidelines](http://www.plosbiology.org/article/info:doi/10.1371/journal.pbio.1000412) for reporting work involving animal research. Where applicable, authors should refer to any relevant reporting standards documents in this form.

If you have any questions, please consult our Journal Policies and/or contact us: [editorial@elifesciences.org](mailto:editorial@elifesciences.org).

**Sample-size estimation**

- You should state whether an appropriate sample size was computed when the study was being designed
- You should state the statistical method of sample size computation and any required assumptions
- If no explicit power analysis was used, you should describe how you decided what sample (replicate) size (number) to use

Please outline where this information can be found within the submission (e.g., sections or figure legends), or explain why this information doesn’t apply to your submission:

Our study utilizes the full sample of page view and link engagement data for the 32-day study period (March 22, 2019-April 22, 2019).

**Replicates**

- You should report how often each experiment was performed
- You should include a definition of biological versus technical replication
- The data obtained should be provided and sufficient information should be provided to indicate the number of independent biological and/or technical replicates
- If you encountered any outliers, you should describe how these were handled
- Criteria for exclusion/inclusion of data should be clearly stated
- High-throughput sequence data should be uploaded before submission, with a private link for reviewers provided (these are available from both GEO and ArrayExpress)

Please outline where this information can be found within the submission (e.g., sections or figure legends), or explain why this information doesn’t apply to your submission:

Due to the nature of our study, we did not conduct any replicates.

**Statistical reporting**

- Statistical analysis methods should be described and justified
- Raw data should be presented in figures whenever informative to do so (typically when N per group is less than 10)
- For each experiment, you should identify the statistical tests used, exact values of N, definitions of center, methods of multiple test correction, and dispersion and precision measures (e.g., mean, median, SD, SEM, confidence intervals; and, for the major substantive results, a measure of effect size (e.g., Pearson's r, Cohen's d)
- Report exact p-values wherever possible alongside the summary statistics and 95% confidence intervals. These should be reported for all key questions and not only when the p-value is less than 0.05.

Please outline where this information can be found within the submission (e.g., sections or figure legends), or explain why this information doesn’t apply to your submission:

In our method section under analysis (pages 5-6), we describe and justify the statistical analyses conducted, including the statistical tests used. The statistical tests and the code utilized are reported in the project’s publicly accessible Github repository (<https://github.com/ryanmax/wiki-citation-usage/>) in the Jupyter notebook labeled: Overview of Event Data.

(For large datasets, or papers with a very large number of statistical tests, you may upload a single table file with tests, Ns, etc., with reference to sections in the manuscript.)

**Group allocation**

- Indicate how samples were allocated into experimental groups (in the case of clinical studies, please specify allocation to treatment method); if randomization was used, please also state if restricted randomization was applied
- Indicate if masking was used during group allocation, data collection and/or data analysis

Please outline where this information can be found within the submission (e.g., sections or figure legends), or explain why this information doesn’t apply to your submission:

This study compares the behaviors of two groups of readers, based on what part of Wikipedia they were reading over the course of the study period, whether (a) the pages managed by WikiProject Medicine or (b) the pages in the rest of Wikipedia. We have described how we determined WikiProject Medicine pages and all other pages in Wikipedia on page 4 of the manuscript.

**Additional data files (“source data”)**

- We encourage you to upload relevant additional data files, such as numerical data that are represented as a graph in a figure, or as a summary table
- Where provided, these should be in the most useful format, and they can be uploaded as “Source data” files linked to a main figure or table
- Include model definition files including the full list of parameters used
- Include code used for data analysis (e.g., R, MatLab)
- Avoid stating that data files are “available upon request”

Please indicate the figures or tables for which source data files have been provided:

With the approval and support of the Wikimedia Foundation (WMF), we accessed the data presented in this study via the [Wikimedia’s Event Logging](https://www.mediawiki.org/wiki/Extension:EventLogging) system and the production MediaWiki database. Due to WMF data policies intended to protect the privacy of readers and editors, all of the data collected and analyzed has had to remain within their protected data environment. Therefore, the data is not publicly available. Researchers may submit a request for the data to the [WMF research team](https://www.mediawiki.org/wiki/Wikimedia_Research/Research_and_Data). The code utilized to collect and analyze the data, however, is organized and made publicly available in a collated series of Jupyter notebooks at: (<https://github.com/ryanmax/wiki-citation-usage/>).

Source data files have been provided for Figure 1 in the project’s publicly accessible Github repository (<https://github.com/ryanmax/wiki-citation-usage/>) in the Jupyter notebook labeled: Page Sizes.
